# 10. An unusual cause for a headache

**DOI:** 10.1093/rap/rky033.002

**Published:** 2018-09-20

**Authors:** Dalia Ludwig, Shahir Hamdulay, Anand Trip, Miren Aizpurua

**Affiliations:** 1 Rheumatology, Northwick Park Hospital, London, UNITED KINGDOM; 2 Neurology, National Hospital for Neurology and Neurosurgery, London, UNITED KINGDOM; 3 Histopathology, National Hospital for Neurology and Neurosurgery, London, UNITED KINGDOM

**Introduction:** Primary central nervous system vasculitis is a rare but important disease to consider in a patient who presents with headache, cognitive impairment and neurological deficit in the absence of systemic disease. As rheumatologists central nervous system vasculitis in the context of systemic inflammatory diseases is well recognised however in the absence of systemic disease a high index of suspicion is required to make the correct diagnosis with relevant investigations to exclude mimics.

**Case description:** We present a twenty-nine year old male IT consultant who is normally fit and well with no relevant past medical history. In January 2014 he presented to the emergency department with numbness of the right arm and face with word finding difficulties. This was preceded by a one week history of intermittent severe frontal headaches lasting up to thirty minutes with vomiting. There was no photophobia, neck stiffness or fever. There was no identifiable trigger, no recent travel and he denied recreational drug use. On examination there was no focal neurology identified and the patient was discharged with an urgent outpatient MRI brain. A provisional diagnosis of migraine was made. One week later he represented with a persistent mild headache, vomiting, facial droop and severe ataxia with drowsiness. He was afebrile with no neck stiffness or photophobia. Examination demonstrated a left sided facial droop, left hemiparesis, sensory loss and left sided neglect. Tone was normal, reflexes symmetrical and brisk with flexor planter responses. He had slow, slurred speech with word finding difficulties. Investigations and results from this admission are summarised in Table 1. A working diagnosis of demyelinating disease was made and he was treated with intravenous methylprednisolone (1 g X3) and then commenced on a weaning dose of prednisolone (60 mg OD). Symptoms and signs rapidly improved and he was discharged. The patient then left the region and was lost to follow-up but then re-presented with similar symptoms in April 2014 to the Edinburgh Royal Infirmary. A repeat MRI brain confirmed progression of multiple infratentorial T2 white matter lesions and MRA showed no evidence of vasculitis. Lumbar puncture revealed a lymphocytic CSF and protein was elevated at 0.85 g/L. Culture and cytology were negative. CT chest abdomen and pelvis, bone marrow aspirate, PET and gallium scans were all normal. He was retreated with prednisolone 60 mg OD with resolution of symptoms but again was lost to follow-up. The working diagnosis at this time was relapsing remitting steroid-responsive lymphocytic meningoencephalitis. Differentials included primary angiitis of the central nervous system (PACNS) and CLIPPERS (chronic lymphocytic inflammation with pontine perivascular enhancement responsive to steroids). Three years later, in June 2017, the patient re-presented with a right retro-orbital headache and confusion. On admission he had left sided hemiparesis and slurred speech. A CT head showed multiple areas of low attenuation consistent with the MRI brain from 2014 with the addition of a possible new right frontal-parietal low attenuation lesion. With the clinical presentation and CT findings the patient was thrombolysed for an acute right total anterior stroke but then became acutely agitated with a decline in GCS from 15 to 6. The patient was intubated, treated for meningoencephalitis and loaded empirically with phenytoin. Salient findings on admission were a normocytic anaemia, lymphopaenia, CRP less than 0.6 mg/dL and ESR 6 mm/hr. Urine toxicology was negative. Anti-neuronal antibodies and Brucella serology were negative. CSF analysis was again predominantly lymphocytic with a protein of 0.49 g/L. Culture was negative as were oligoclonal bands. MRI showed progression of T2 white matter hyperintensity. MRA showed normal vessels. The appearances were reported to be more suggestive of progressive leukoencephalopathy secondary to toxic/metabolic (including genetic) or mitochondrial disease rather than vasculitis. Electroencephalogram reported right posterior temporal slowing which was non-specific with no epileptiform activity. Neuropsychometry showed a mild degree of anterior and subcortical compromise. In summary this patient had a relapsing, remitting neuroinflammatory syndrome with encephalopathy and focal neurology in the absence of systemic inflammation. The CSF was predominantly lymphocytic with mildly raised protein and progressive white matter changes were present on MRI. These episodes were acutely steroid responsive with resolution of neurology. Differentials included neurosarcoidosis, intravascular lymphoma, PACNS and several rare inherited small vessel and mitochondrial diseases which were subsequently excluded by a normal muscle and skin biopsy. In view of the broad differential the patient had a brain biopsy. Histology showed multifocal perivascular inflammatory changes in the white matter with histiocytes and lymphocytes forming ill-defined non-necrotising granulomatous structures with no evidence of fibrinoid necrosis or vessel wall disruption. The mild leptomeningeal component compared to more severe white matter involvement made a diagnosis of neurosarcoidosis less likely. Microorganisms were not identified and there were no signs of underlying malignancy. Features were in keeping with perivascular inflammation with granulomata, suggestive of primary angiitis of the central nervous system (PACNS). The patient was treated with a tapering dose of prednisolone and cyclophosphamide, as per the European Vasculitis Society guidelines 2009 for remission induction. For maintenance therapy he has been prescribed mycophenolate mofetil. There has been resolution of neurology and no further relapses reported to date. The patient is working full time and back to full physical health.

**Discussion:** The clinical presentation, laboratory findings, imaging, skin and muscle histology were inconclusive in providing a diagnosis. Subjecting the patient to strong immunosuppressive therapy without having a definitive diagnosis led the team to persist in gaining histology from the primary lesion, the brain. Brain histology provided the evidence needed to make the right diagnosis. This is an interesting case as brain biopsy is the gold standard for the diagnosis of PACNS but rarely performed. The range of investigations show how we arrived at the diagnosis and how the mimics of this disease were excluded during this process. We have learnt the different ways PACNS can present and more importantly its mimics and how to investigate for these in a timely stepwise process. We have learnt how negative results are as important as positive ones when faced with a challenging case. For example the presence of a normal inflammatory response raised the suspicion from the start that this was not a systemic illness. We have also learnt the importance of involving different specialists in challenging cases. In this case the expertise of neurologists, haematologists, infectious diseases and histopathologists were called upon. We have also learnt the different ways PACNS can appear on brain imaging and if the diagnosis is uncertain then brain biopsy should be sought. This case report raises the discussion as to whether, as a speciality, we should be referring more of our patients for brain biopsy when the diagnosis is unclear particularly in those patients who have an absence of systemic disease.

**Key Learning Points:** The main learning points in this case are to appreciate PACNS is a rare disease but has a long list of mimics and some of those will fall under the remit of rheumatology, therefore we should be aware of its presentation and how to investigate in a way that carefully excludes the possible differentials. We have learnt the importance of retaining a high index of suspicion when faced with patients presenting with neurological signs and symptoms particularly when there is an absence of significant systemic disease and which important investigations are required to achieve the correct diagnosis. The key learning objective is to highlight PACNS as a possible differential in patients with neurological involvement where vasculitis is a diagnostic consideration but where there is an absence of systemic features one would normally expect to find. Further objectives include reviewing the different ways PACNS can present itself clinically and radiologically and if the diagnosis is in doubt to follow a series of investigations which may lead one to the gold standard that is brain biopsy. Finally, this case shows certain findings that are common to PACNS for example lack of ESR/CRP response, lymphopaenia, lymphocytic CSF with mildly elevated protein and positive oligoclonal bands which are absent in serum and non-specific white matter changes on brain imaging. These findings may help to point towards a diagnosis of PACNS and are important for the rheumatologist to be aware of when investigating patients with possible vasculitis and neurological involvement.

**70.** Table 1: Investigations and results

Laboratory	Radiology	CSF
Haemoglobin 150 g/L, normocytic Platelet count 172 (x10^9^/L) White cell count 9.2 (x10^9^/L) Neutrophil 8.5 (x10^9^/L) Lymphocyte 0.5 (x10^9^/L) Renal/Liver/Bone profile – normal CRP 1 mg/dL ESR 2 mm/hr ANA/ENA/ANCA/RF - negative C3/C4 - normal Immunoglobulins - normal Anti-cardiolipin IgG/IgM - negative Serum ACE – negative Borrelia Burgdoferi IgG/IgM - negative Hep A/B/C and HIV - negative	MRI Brain - widespread ill defined periventricular white matter T2 hyper-intensities and a number of focal white matter lesions in the cerebellum. MRI (GAD) - No abnormal enhancement.	Clear colourless Acellular Culture negative including mycobacterial species Glucose 3.9 mmol/L Lac 1.9 mmol/L Protein 0.43 g/L Oligoclonal bands - positive Serum bands - negative

**Disclosure: D. Ludwig:** None. **S. Hamdulay:** None. **A. Trip:** None. **M. Aizpurua:** None.

